# Epidemiological survey of patients with pustular psoriasis in the Japanese Society for Psoriasis Research from 2017 to 2020

**DOI:** 10.1111/1346-8138.16583

**Published:** 2022-09-24

**Authors:** Koji Kamiya, Mamitaro Ohtsuki

**Affiliations:** ^1^ Department of Dermatology Jichi Medical University Shimotsuke Japan

**Keywords:** dermatology, epidemiology, Japan, Japanese Society for Psoriasis Research, pustular psoriasis

## Abstract

The Japanese Society for Psoriasis Research (JSPR) has been conducting annual epidemiological surveys of patients with pustular psoriasis in Japan since 2017. This study aimed to conduct a recent epidemiological analysis of patients with pustular psoriasis who were enrolled in the JSPR from 2017 to 2020. A total of 291 patients from 131 medical institutions were enrolled, of which 47.4% (138 cases) were males and 52.6% (153 cases) were females. The mean ± standard deviation (SD) age of the patients was 57.4 ± 20.3 years (males, 61.2 ± 17.3 years; females, 54.1 ± 22.1 years). The mean ± SD age of the patients at disease onset was 48.5 ± 22.5 years (males, 50.8 ± 20.6 years; females, 46.4 ± 24.0 years). The types of pustular psoriasis observed included the von Zumbusch type (59.8%), annular pustular psoriasis (8.2%), impetigo herpetiformis (6.5%), and acrodermatitis continua of Hallopeau (4.8%), of which, the majority of the patients with impetigo herpetiformis were female. Among the patients, 58.4% were treated with oral medications and 44.0% were treated with biologics. The most common oral medication prescribed was etretinate (52.4%), followed by corticosteroids (24.7%) and cyclosporin (22.9%). The most common biologics used were IL‐17 inhibitors (ixekizumab [28.1%] and secukinumab [22.7%]), followed by tumor necrosis factor (TNF) inhibitors (infliximab [15.6%]) and IL‐23 inhibitors (guselkumab [14.8%] and risankizumab [10.2%]). This survey thus provides new and significant information regarding the recent perspective of pustular psoriasis, such as patient characteristics and treatment trends, in Japan.

## 1 INTRODUCTION

Psoriasis is a common chronic inflammatory skin disease that can present at any age.^1^ Various risk factors and comorbidities are known to interact with each other and elicit disease.^2^ The Japanese Society for Psoriasis Research (JSPR) has been conducting annual epidemiological surveys of patients with psoriasis since 1982.^3^, ^4^, ^5^, ^6^ Kawada et al.^3^ reported 28 628 cases that were enrolled during 1982–2001, while Takahashi et al.^4^ reported 11 631 cases during 2002–2008. Ito et al. reported 9290 cases during 2009–2012^5^ and Kamiya et al.^6^ reported 15 287 cases during 2013–2018. These studies have provided significant information on psoriasis, such as changes in the age at onset, comorbidities, and treatment trends.

Pustular psoriasis is a rare, severe phenotype. Recent clinical, histological, and genetic evidence suggests that generalized pustular psoriasis (GPP) is a clinical entity distinct from plaque psoriasis.^7^ In Japan, Ohkawara et al. reported the characteristics of 541 GPP cases in 1996,^8^ while Ohata et al.^9^ reported 104 cases in 2021. Ozawa et al.^10^ reported treatments and outcomes of 385 GPP cases in 1999, while Miyachi et al.^11^ reported 1516 cases in 2021. In addition, the Japanese guidelines for the management and treatment of GPP were published in 2018.^12^ However, till date, only few studies have been conducted to determine the characteristics of pustular psoriasis cases and treatment trends in Japan. Since 2017, the JSPR has conducted annual epidemiological surveys of patients with pustular psoriasis. Thus, this study aimed to conduct a recent epidemiological analysis of patients with pustular psoriasis who were enrolled in the JSPR from 2017 to 2020.

## 2 METHODS

The JSPR partners with medical institutions throughout Japan. It uses its own questionnaire to perform annual surveys and collect data on newly diagnosed pustular psoriasis cases (from April of the previous year to March of the survey year). A total of 131 medical institutions participated in the surveys for the present study, conducted from 2017 to 2020. The survey was designed to acquire information about patient characteristics, lifestyle habits, disease severity and subtype, family history, past history and comorbidities, exacerbating factors, focal infection, distribution of lesions, and current treatments. Only data from the completed surveys were included. This study was approved by the Ethical Committee of Jichi Medical University, the central institute that oversees the entire survey.

## 3 RESULTS

### 3.1 Population

**FIGURE 1:**
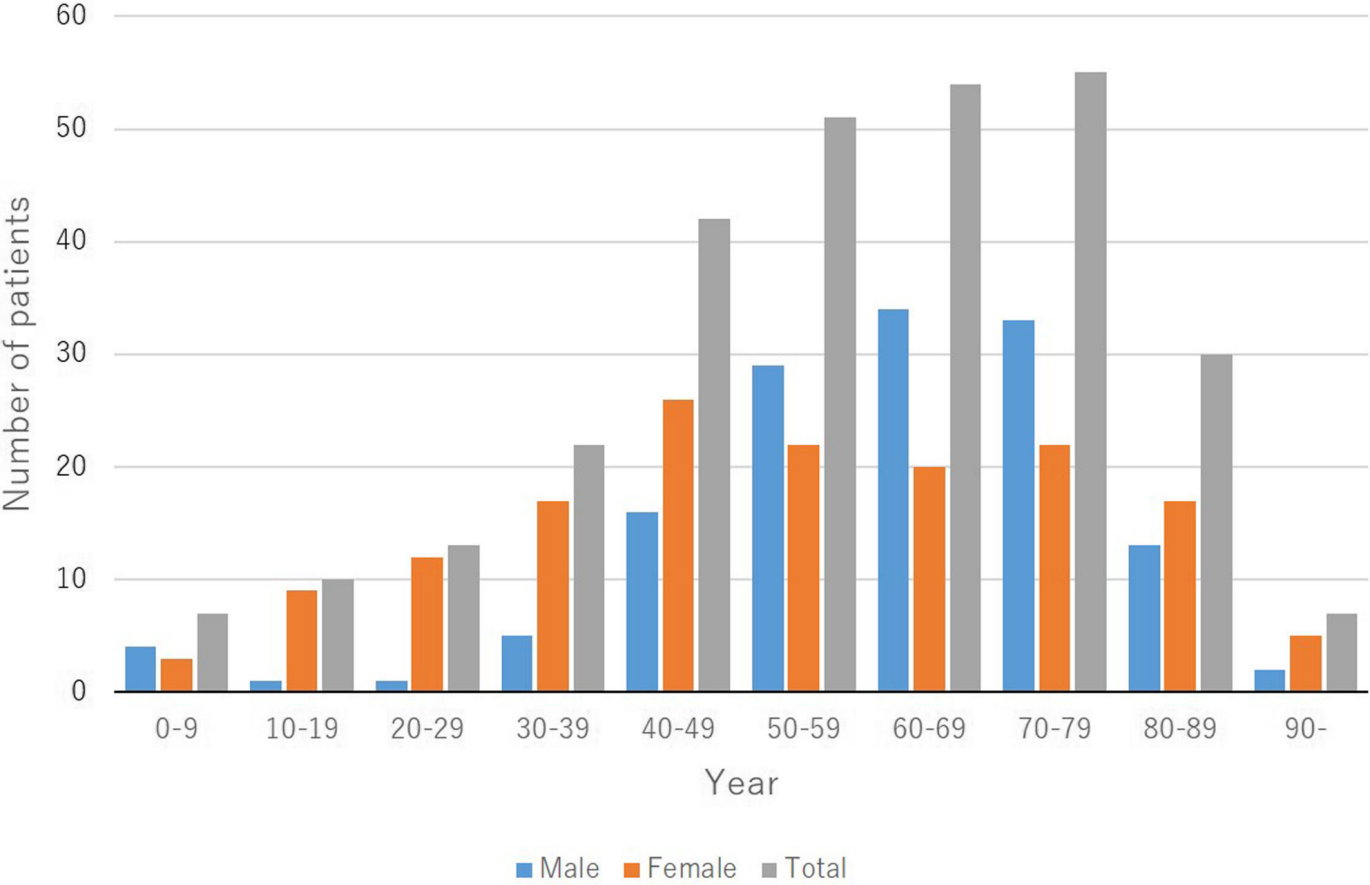
Age and sex distribution

A total of 291 patients were enrolled from 2017 to 2020, of which 47.4% (138 cases) were male and 52.6% (153 cases) were female. The age at which patients were initially diagnosed varied from 1 year to 96 years. The mean ± standard deviation (SD) age of the patients was 57.4 ± 20.3 years (males, 61.2 ± 17.3 years; females, 54.1 ± 22.1 years). The age distributions were as follows: 7 patients aged 0–9 years (2.4%; 4 boys [2.9%] and 3 girls [2.0%]), 10 aged 10–19 years (3.4%; 1 boy [0.7%] and 9 girls [5.9%]), 13 aged 20–29 years (4.5%; 1 man [0.7%] and 12 women [7.8%]), 22 aged 30–39 years (7.6%; 5 men [3.6%] and 17 women [11.1%]), 42 aged 40–49 years (14.4%; 16 men [11.6%] and 26 women [17.0%]), 51 aged 50–59 years (17.5%; 29 men [21.0%] and 22 women [14.4%]), 54 aged 60–69 years (18.6%; 34 men [24.6%] and 20 women [13.1%]), 55 aged 70–79 years (18.9%; 33 men [23.9%] and 22 women [14.4%]), 30 aged 80–89 years (10.3%; 13 men [9.4%] and 17 women [11.1%]), and 7 aged 90 years or older (2.4%; 2 men [1.4%] and 5 women [3.3%]) (Figure 1).

### 3.2 Age at onset

**FIGURE 2:**
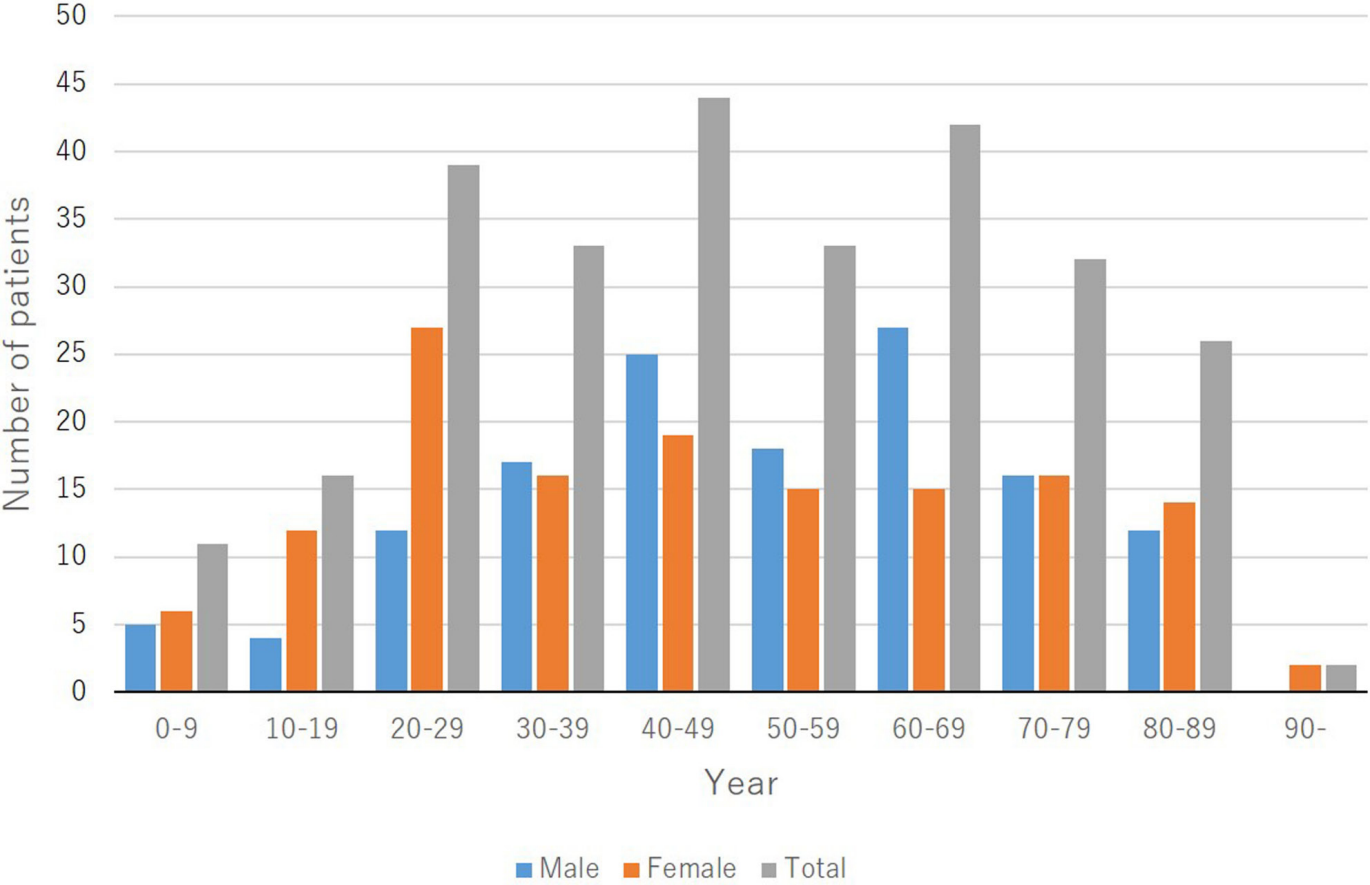
Age at onset

The mean ± SD age of the patients at disease onset was 48.5 ± 22.5 years (males, 50.8 ± 20.6 years; females, 46.4 ± 24.0 years). The age at disease onset was 0–9 years for 11 patients (4.0%; 5 boys [3.7%] and 6 girls [4.2%]), 10–19 years for 16 patients (5.8%; 4 boys [2.9%] and 12 girls [8.5%]), 20–29 years for 39 patients (14.0%; 12 men [8.8%] and 27 women [19.0%]), 30–39 years for 33 patients (11.9%; 17 men [12.5%] and 16 women [11.3%]), 40–49 years for 44 patients (15.8%; 25 men [18.4%] and 19 women [13.4%]), 50–59 years for 33 patients (11.9%; 18 men [13.2%] and 15 women [10.6%]), 60–69 years for 42 patients (15.1%; 27 men [19.9%] and 15 women [10.6%]), 70–79 years for 32 patients (11.5%; 16 men [11.8%] and 16 women [11.3%]), 80–89 years for 26 patients (9.4%; 12 men [8.8%] and 14 women [9.9%]), and 90 years and older for 2 patients (0.7%; 2 women [1.4%]) (Figure 2).

### 3.3 Patient characteristics

The mean ± SD height (cm) of the patients was 157.9 ± 16.7 (males, 163.6 ± 17.8; females, 152.2 ± 13.3) (Table 1). The mean ± SD weight (kg) was 58.8 ± 17.5 (males, 63.6 ± 17.3; females, 54.4 ± 16.6). The mean ± SD body mass index (BMI) was 22.8 ± 4.9 (males, 23.0 ± 4.4; females, 22.7 ± 5.3). Some patients consumed alcohol (100 patients [34.4%]; 61 men [44.2%] and 39 women [25.5%]) and/or smoked (87 patients [29.9%]; 60 men (43.5%) and 27 women [17.6%]). Some patients had a history of atopic dermatitis (11 patients [3.8%]; 5 men [3.6%] and 6 women [3.9%]), bronchial asthma (12 cases [4.1%]; 4 men [2.9%] and 8 women [5.2%]), hay fever (19 cases [6.5%]; 11 men [8.0%] and 8 women [5.2%]), and visceral malignancies (35 cases [12.0%]; 22 men [15.9%] and 13 women [8.5%]). A total of 116 patients (39.9%; 68 men [49.3%] and 48 women [31.4%]) had a history of psoriasis vulgaris (PsV), while 36 patients (12.4%; 17 men [12.3%] and 19 women [12.4%]) had a history of psoriatic arthritis (PsA). The mean ± SD incidence of symptomatic worsening was 1.5 ± 1.8 (males, 1.6 ± 2.4; females, 1.4 ± 0.9).

**TABLE 1:** Patient characteristics

	Male	Female	All
Number of patients	138	153	291
Height (cm), mean ± SD	163.6 ± 17.8	152.2 ± 13.3	157.9 ± 16.7
Weight (kg), mean ± SD	63.6 ± 17.3	54.4 ± 16.6	58.8 ± 17.5
BMI, mean ± SD	23.0 ± 4.4	22.7 ± 5.3	22.8 ± 4.9
Lifestyle habits			
Alcohol	61 (44.2%)	39 (25.5%)	100 (34.4%)
Smoking	60 (43.5%)	27 (17.6%)	87 (29.9%)
Past history			
Atopic dermatitis	5 (3.6%)	6 (3.9%)	11 (3.8%)
Bronchial asthma	4 (2.9%)	8 (5.2%)	12 (4.1%)
Hay fever	11 (8.0%)	8 (5.2%)	19 (6.5%)
Malignancies	22 (15.9%)	13 (8.5%)	35 (12.0%)
Psoriasis vulgaris	68 (49.3%)	48 (31.4%)	116 (39.9%)
Psoriatic arthritis	17 (12.3%)	19 (12.4%)	36 (12.4)

Abbreviations: BMI, body mass index; SD, standard deviation.

### 3.4 Severity and subtype

The percentage of patients with <5%, 5%–10%, 10%–20%, and > 20% of the body surface area (BSA) affected was 10.7% (males, 8.0%; females, 13.1%), 10.0% (males, 8.7%; females, 11.1%), 12.0% (males, 10.9%; females, 13.1%), and 57.0% (males, 60.1%; females, 54.2%), respectively (Table 2). The types of psoriasis observed included the von Zumbusch type (59.8%; males, 61.6%; females, 58.2%), annular pustular psoriasis (8.2%; males, 8.0%; females, 8.5%), impetigo herpetiformis (6.5%; males, 1.4%; females, 11.1%), and acrodermatitis continua of Hallopeau (4.8%; males, 4.3%; females, 5.2%). Arthralgia, fever, and itchiness were observed in 20.6% (males, 22.5%; females, 19.0%), 34.7% (males, 35.5%; females, 34.0%), and 56.4% (males, 58.0%; females, 54.9%) of patients, respectively.

**TABLE 2:** Severity and subtype

	Male	Female	All
Severity			
BSA <5%	11 (8.0%)	20 (13.1%)	31 (10.7%)
BSA 5%–10%	12 (8.7%)	17 (11.1%)	29 (10.0%)
BSA 10%–20%	15 (10.9%)	20 (13.1%)	35 (12.0%)
BSA > 20%	83 (60.1%)	83 (54.2%)	166 (57.0%)
Unknown	17 (12.3%)	13 (8.5%)	30 (10.3%)
Subtype			
von Zumbusch type	85 (61.6%)	89 (58.2%)	174 (59.8%)
Annular pustular psoriasis	11 (8.0%)	13 (8.5%)	24 (8.2%)
Impetigo herpetiformis	2 (1.4%)	17 (11.1%)	19 (6.5%)
Acrodermatitis continua of Hallopeau	6 (4.3%)	8 (5.2%)	14 (4.8%)
Others	4 (2.9%)	5 (3.3%)	9 (3.1%)
Unknown	32 (23.2%)	25 (16.3%)	57 (19.6%)

Abbreviation: BSA, body surface area.

### 3.5 Family history

Twelve patients (4.1%) had a family history of psoriasis (4 men [2.9%] and 8 women [5.2%]). The affected family members included fathers (2 women [25.0%]), mothers (1 woman [12.5%]), children (1 woman [12.5%]), siblings (2 men [50.0%] and 1 woman [12.5%]), and others (1 man [25.0%] and 5 women [62.5%]). In addition, 5 patients (1.7%) had a family history of palmoplantar pustulosis (PPP) (2 men [1.4%] and 3 women [2.0%]). The affected family members included children (1 woman [33.3%]) and others (1 man [50.0%] and 2 women [66.7%]).

### 3.6 Past history and comorbidities

A total of 196 patients (67.4%) had a history of comorbidities (104 men [75.4%] and 92 women [60.1%]). The patients' past history and comorbidities included hypertension (78 cases [39.8%]; 47 men [45.2%] and 31 women [33.7%]), dyslipidemia (47 cases [24.0%]; 27 men [26.0%] and 20 women [21.7%]), diabetes mellitus (45 cases [23.0%]; 31 men [29.8%] and 14 women [15.2%]), hyperuricemia (13 cases [6.6%]; 13 men [12.5%]), cardiovascular disease (14 cases [7.1%]; 9 men [8.7%] and 5 women [5.4%]), and cerebrovascular disease (15 cases [7.7%]; 7 men [6.7%] and 8 women [8.7%]) (Table 3).

**TABLE 3:** Patient history and comorbidities

	Male	Female	All
Hypertension	47 (45.2%)	31 (33.7%)	78 (39.8%)
Dyslipidemia	27 (26.0%)	20 (21.7%)	47 (24.0%)
Diabetes mellitus	31 (29.8%)	14 (15.2%)	45 (23.0%)
Hyperuricemia	13 (12.5%)	0 (0%)	13 (6.6%)
Cardiovascular disease	9 (8.7%)	5 (5.4%)	14 (7.1%)
Cerebrovascular disease	7 (6.7%)	8 (8.7%)	15 (7.7%)

### 3.7 Exacerbating factors

Eighty patients (27.5%) had exacerbating factors (36 men [26.1%] and 44 women [28.8%]). The exacerbating factors included infection (21 patients [26.3%]; 13 men [36.1%] and 8 women [18.2%]), stress (20 cases [25.0%]; 10 men [27.8%] and 10 women [22.7%]), certain drugs (18 cases [22.5%]; 7 men [19.4%] and 11 women [25.0%]), pregnancy (12 cases [15.0%]; 12 women [27.3%]), certain seasons (6 cases [7.5%]; 4 men [11.1%] and 2 women [4.5%]), fatigue (6 cases [7.5%]; 3 men [8.3%] and 3 women [6.8%]), and sun exposure (1 case [1.3%]; 1 man [2.8%]) (Table 4).

**TABLE 4:** Exacerbating factors

	Male	Female	All
Infection	13 (36.1%)	8 (18.2%)	21 (26.3%)
Stress	10 (27.8%)	10 (22.7%)	20 (25.0%)
Drug	7 (19.4%)	11 (25.0%)	18 (22.5%)
Pregnancy	0 (0%)	12 (27.3%)	12 (15.0%)
Seasonal factors	4 (11.1%)	2 (4.5%)	6 (7.5%)
Fatigue	3 (8.3%)	3 (6.8%)	6 (7.5%)
Sun exposure	1 (2.8%)	0 (0%)	1 (1.3%)

### 3.8 Focal infection

A total of 34 patients (11.7%) had some kind of focal infection (17 men [12.3%] and 17 women [11.1%]). Focal infections included odontogenic infections (18 cases [52.9%]; 10 men [58.8%] and 8 women [47.1%]), tonsillitis (7 cases [20.6%]; 4 men [23.5%] and 3 women [17.6%]), and sinusitis (4 cases [11.8%]; 3 men [17.6%] and 1 woman [5.9%]).

### 3.9 Distribution of skin lesions at the first examination

**FIGURE 3:**
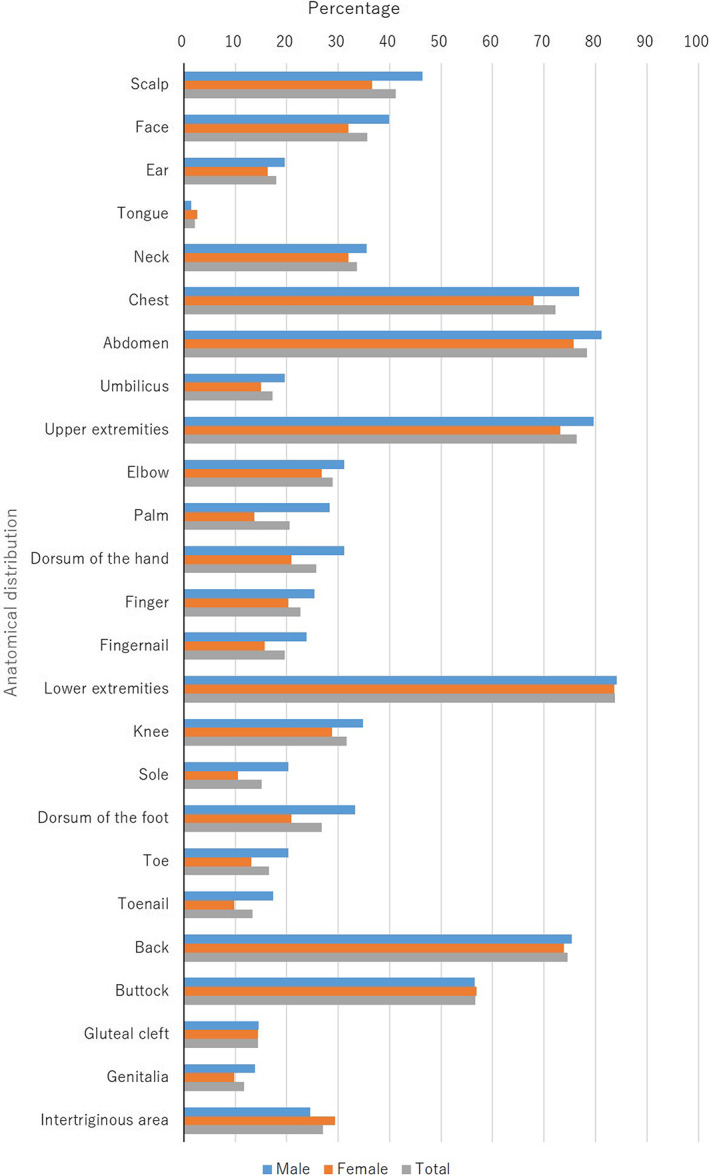
Anatomical distribution of the skin lesions during the first examination

The skin lesions were located on the scalp (41.2%; males, 46.4%; females, 36.6%), the face (35.7%; males, 39.9%; females, 32.0%), the ear (17.9%; males, 19.6%; females, 16.3%), the tongue (2.1%; males, 1.4%; females, 2.6%), the neck (33.7%; males, 35.5%; females, 32.0%), the chest (72.2%; males, 76.8%; females, 68.0%), the abdomen (78.4%; males, 81.2%; females, 75.8%), the umbilicus (17.2%; males, 19.6%; females, 15.0%), the upper extremities (76.3%; males, 79.7%; females, 73.2%), the elbow (28.9%; males, 31.2%; females, 26.8%), the palm (20.6%; males, 28.3%; females, 13.7%), the dorsum of the hand (25.8%; males, 31.2%; females, 20.9%), the finger (22.7%; males, 25.4%; females, 20.3%), the fingernail (19.6%; males, 23.9%; females, 15.7%), the lower extremities (83.8%; males, 84.1%; females, 83.7%), the knee (31.6%; males, 34.8%; females, 28.8%), the sole (15.1%; males, 20.3%; females, 10.5%), the dorsum of the foot (26.8%; males, 33.3%; females, 20.9%), the toe (16.5%; males, 20.3%; females, 13.1%), the toenail (13.4%; males, 17.4%; females, 9.8%), the back (74.6%; males, 75.4%; females, 73.9%), the buttocks (56.7%; males, 56.5%; females, 56.9%), the gluteal cleft (14.4%; males, 14.5%; females, 14.4%), the genitalia (11.7%; males, 13.8%; females, 9.8%), and the intertriginous area (27.1%; males, 24.6%; females, 29.4%) at the first examination (Figure 3).

### 3.10 Treatments

**FIGURE 4:**
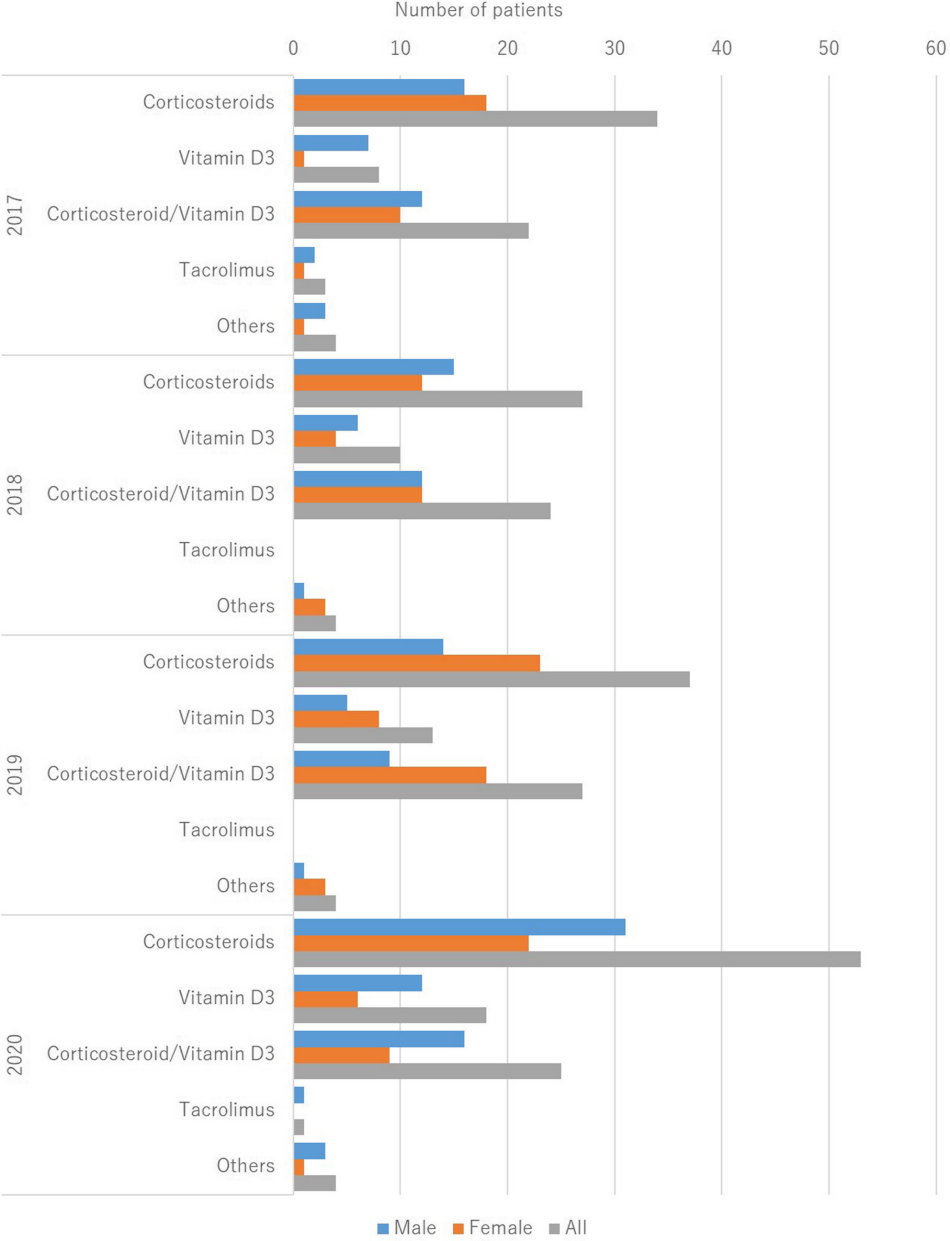
Treatment trends in topical therapy

**FIGURE 5:**
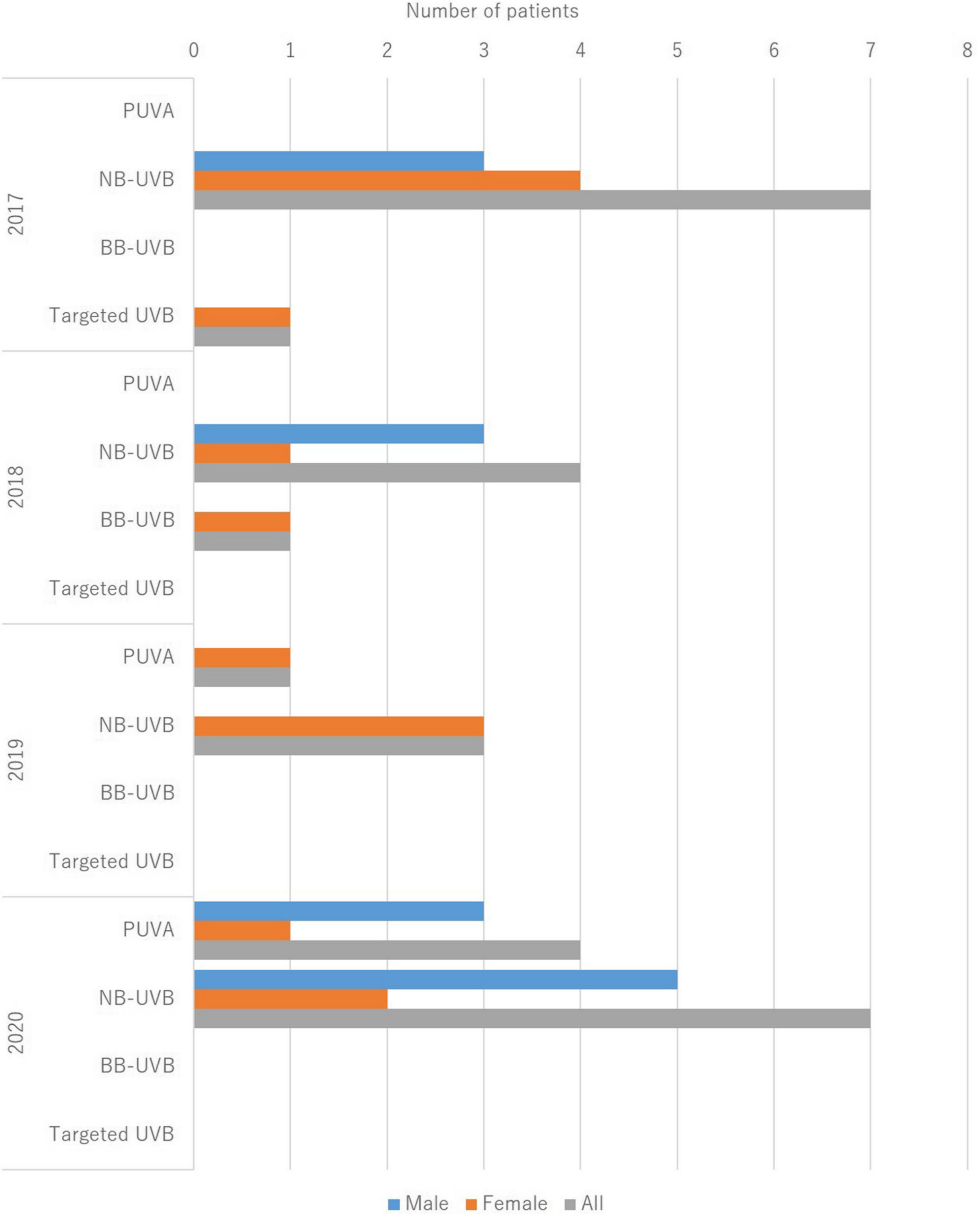
Treatment trends in phototherapy. BB‐UVB, broadband ultraviolet B; NB‐UVB, narrowband ultraviolet B; PUVA, psoralen ultraviolet A.

**FIGURE 6:**
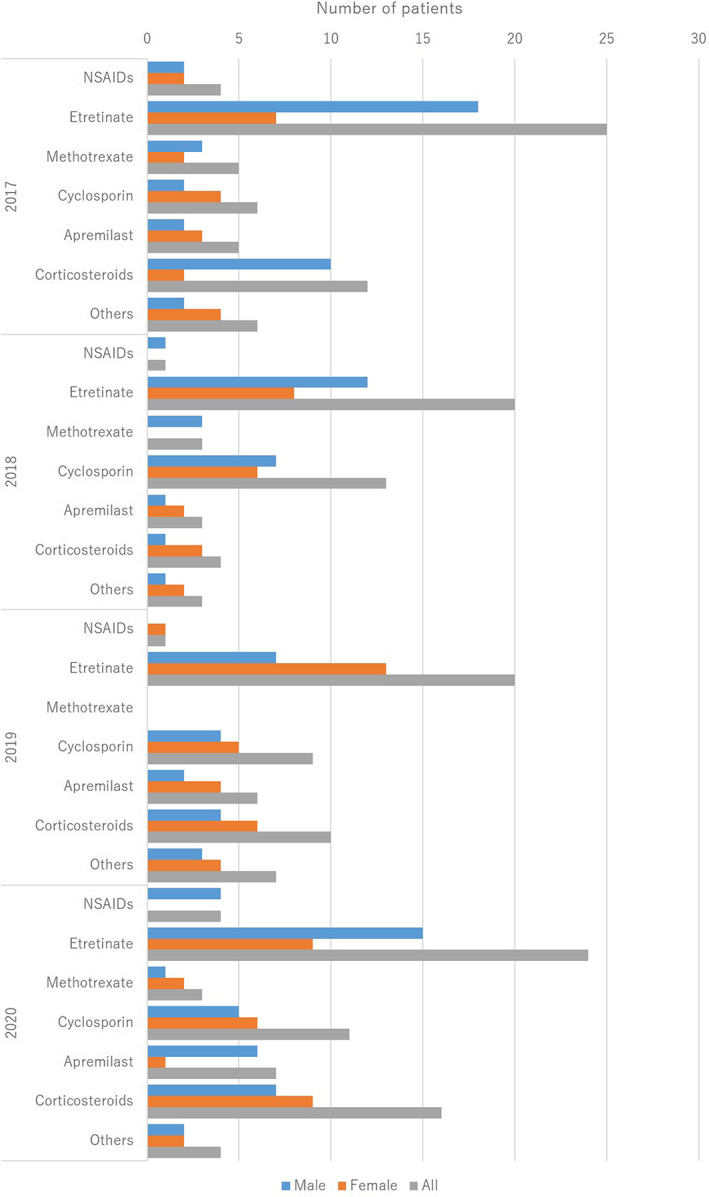
Treatment trends in the oral medication. NSAIDs, non‐steroidal anti‐inflammatory drugs.

**FIGURE 7:**
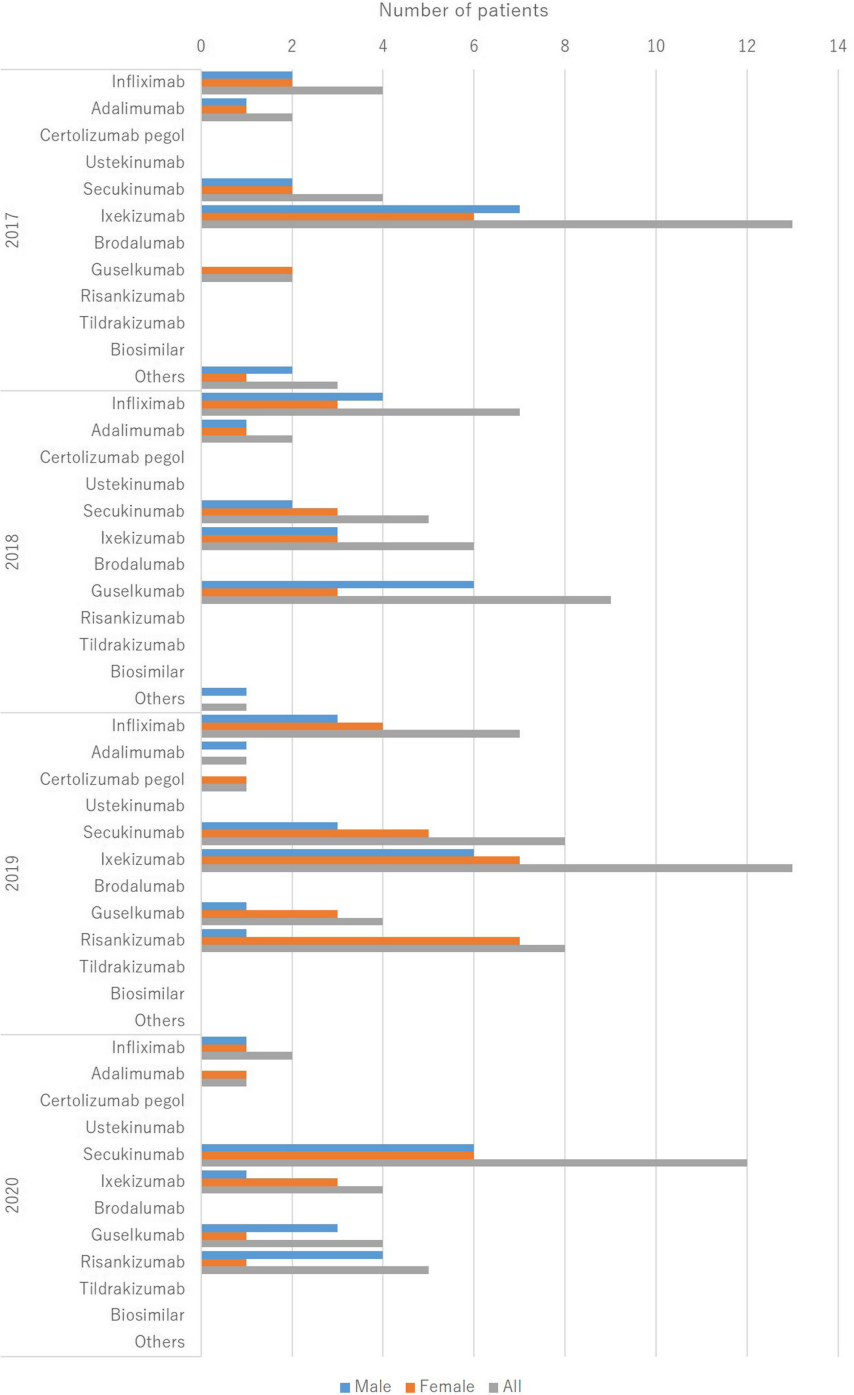
Treatment trends in the biologics

The treatments are summarized in Table 5 and the treatment trends are shown in (Figure 4, 5, 6, 7). A total of 225 patients (77.3%) received topical therapy (109 men [79.0%] and 116 women [75.8%]). Topical therapies included corticosteroids (151 cases [67.1%]; 76 men [69.7%] and 75 women [64.7%]), vitamin D3 (49 cases [21.8%]; 30 men [27.5%] and 19 women [16.4%]), corticosteroid/vitamin D3 combinations (98 cases [43.6%]; 49 men [45.0%] and 49 women [42.2%]), tacrolimus (4 cases [1.8%]; 3 men [2.8%] and 1 woman [0.9%]), and others (16 cases [7.1%]; 8 men [7.3%] and 8 women [6.9%]).

**TABLE 5:** Treatments for psoriasis

	Male	Female	All
Topical therapy	109 (79.0%)	116 (75.8%)	225 (77.3%)
Corticosteroids	76 (69.7%)	75 (64.7%)	151 (67.1%)
Vitamin D3	30 (27.5%)	19 (16.4%)	49 (21.8%)
Corticosteroid/vitamin D3	49 (45.0%)	49 (42.2%)	98 (43.6%)
Tacrolimus	3 (2.8%)	1 (0.9%)	4 (1.8%)
Others	8 (7.3%)	8 (6.9%)	16 (7.1%)
Phototherapy	14 (10.1%)	14 (9.2%)	28 (9.6%)
PUVA	3 (21.4%)	2 (14.3%)	5 (17.9%)
NB‐UVB	11 (78.6%)	10 (71.4%)	21 (75.0%)
BB‐UVB	0 (0%)	1 (7.1%)	1 (3.6%)
Targeted UVB	0 (0%)	1 (7.1%)	1 (3.6%)
Systemic therapy			
Oral medication	91 (65.9%)	79 (51.6%)	170 (58.4%)
Etretinate	52 (57.1%)	37 (46.8%)	89 (52.4%)
Methotrexate	7 (7.7%)	4 (5.1%)	11 (6.5%)
Cyclosporin	18 (19.8%)	21 (26.6%)	39 (22.9%)
Apremilast	11 (12.1%)	10 (12.7%)	21 (12.4%)
Corticosteroids	22 (24.2%)	20 (25.3%)	42 (24.7%)
NSAIDs	7 (7.7%)	3 (3.8%)	10 (5.9%)
Others	8 (8.8%)	12 (15.2%)	20 (11.8%)
Biologics	61 (44.2%)	67 (43.8%)	128 (44.0%)
Infliximab	10 (16.4%)	10 (14.9%)	20 (15.6%)
Adalimumab	3 (4.9%)	3 (4.5%)	6 (4.7%)
Certolizumab pegol	0 (0%)	1 (1.5%)	1 (0.8%)
Ustekinumab	0 (0%)	0 (0%)	0 (0%)
Secukinumab	13 (21.3%)	16 (23.9%)	29 (22.7%)
Ixekizumab	17 (27.9%)	19 (28.4%)	36 (28.1%)
Brodalumab	0 (0%)	0 (0%)	0 (0%)
Guselkumab	10 (16.4%)	9 (13.4%)	19 (14.8%)
Risankizumab	5 (8.2%)	8 (11.9%)	13 (10.2%)
Tildrakizumab	0 (0%)	0 (0%)	0 (0%)
Biosimilar	0 (0%)	0 (0%)	0 (0%)
Others	3 (4.9%)	1 (1.5%)	4 (3.1%)
GMA	9 (6.5%)	18 (11.8%)	27 (9.3%)

Abbreviations: BB‐UVB, broadband ultraviolet B; GMA, granulocyte and monocyte adsorptive apheresis; NB‐UVB, narrowband ultraviolet B; NSAIDs, non‐steroidal anti‐inflammatory drugs; PUVA, psoralen ultraviolet A.

A total of 28 patients (9.6%) received phototherapy (14 men [10.1%] and 14 women [9.2%]). Phototherapy included psoralen ultraviolet A (PUVA) (5 cases [17.9%]; 3 men [21.4%] and 2 women [14.3%]), narrowband (NB)‐UVB (21 cases [75.0%]; 11 men [78.6%] and 10 women [71.4%]), broadband (BB)‐UVB (1 case [3.6%]; 1 woman [7.1%]), and targeted UVB (1 case [3.6%]; 1 woman [7.1%]).

Systemic therapy can be divided into oral medication and biologics. A total of 170 patients (58.4%) were treated with oral medications (91 men [65.9%] and 79 women [51.6%]). Oral medications included etretinate (89 cases [52.4%]; 52 men [57.1%] and 37 women [46.8%]), methotrexate (11 cases [6.5%]; 7 men [7.7%] and 4 women [5.1%]), cyclosporin (39 cases [22.9%]; 18 men [19.8%] and 21 women [26.6%]), apremilast (21 cases [12.4%]; 11 men [12.1%] and 10 women [12.7%]), corticosteroids (42 cases [24.7%]; 22 men [24.2%] and 20 women [25.3%]), non‐steroidal anti‐inflammatory drugs (NSAIDs) (10 cases [5.9%]; 7 men [7.7%] and 3 women [3.8%]), and others (20 cases [11.8%]; 8 men [8.8%] and 12 women [15.2%]). A total of 128 patients (44.0%) were treated with biologics (61 men [44.2%] and 67 women [43.8%]). The biologics included infliximab (20 cases [15.6%]; 10 men [16.4%] and 10 women [14.9%]), adalimumab (6 cases [4.7%]; 3 men [4.9%] and 3 women [4.5%]), certolizumab pegol (1 case [0.8%]; 1 woman [1.5%]), secukinumab (29 cases [22.7%]; 13 men [21.3%] and 16 women [23.9%]), ixekizumab (36 cases [28.1%]; 17 men [27.9%] and 19 women [28.4%]), guselkumab (19 cases [14.8%]; 10 men [16.4%] and 9 women [13.4%]), risankizumab (13 cases [10.2%]; 5 men [8.2%] and 8 women [11.9%]), and others (4 cases [3.1%]; 3 men [4.9%] and 1 woman [1.5%]). Approximately 10.2% of the patients presented with paradoxical reactions. Discontinuation or bioswitch was observed in 29 cases. In patients who received a bioswitch, the most common initial biologics were infliximab (7 cases), followed by adalimumab (5 cases), secukinumab (5 cases), and ixekizumab (4 cases). In patients initially treated with infliximab, the second biologic was adalimumab (3 cases), secukinumab (3 cases), and ixekizumab (1 case). In patients initially treated with adalimumab, the second biologic was ixekizumab (4 cases) and risankizumab (1 case). In patients initially treated with secukinumab, the second biologic was adalimumab (2 cases), ixekizumab (2 cases), and certolizumab pegol (1 case). In patients initially treated with ixekizumab, the second biologic was guselkumab (3 cases) and certolizumab pegol (1 case). Seven patients were treated with more than 3 biologics. The total number of biologics used was 3 (4 cases), 4 (1 case), 5 (1 case), and 6 (1 case). In these cases, the most common initial biologic drug was infliximab (5 cases), among which the second biologics were adalimumab (3 cases) and secukinumab (2 cases). In the present study, none of the patients were treated with ustekinumab, brodalumab, tildrakizumab, or biosimilars. Granulocyte and monocyte adsorption apheresis (GMA) is approved for the treatment of pustular psoriasis, and 27 patients (9.3%) were treated with GMA (9 men [6.5%] and 18 women [11.8%]).

## 4 DISCUSSION

In the present study, 291 patients were enrolled during 2017–2020 from 131 medical institutions, of which 47.4% (138 cases) were male and 52.6% (153 cases) were female. In a Japanese multicenter observational study, a total of 104 cases were enrolled, of which 52.9% were male.^9^ In another retrospective study using the Japanese Medical Data Vision claims database, a total of 348 cases were enrolled, of which 48.5% of the cases were males.^13^ The male: female ratio seems to be almost 1:1 in Japanese patients with pustular psoriasis. In contrast, the male: female ratio was 2.7:1 in a retrospective study analyzing Chinese patients,^14^ and 1:2 in a retrospective study analyzing Malaysian patients.^15^ In Brazil, a public claims database study reported 1458 cases of pustular psoriasis, of which 53% were female.^16^ The predominance of males or females may vary with the country.

In terms of age distribution, the proportion of patients increased gradually and subsequently after the age of 40 years (Figure 1). The peak was observed in the 70–79‐year‐old group and subsequently decreased thereafter. In male patients, the proportion increased gradually and subsequently after the age of 40 years. The peak was observed in the 60–69‐year‐old group and 70–79‐year‐old group. In contrast, the proportion increased gradually, peaked in the 40–49‐year‐old group, and then gradually decreased in the female patients. In a retrospective study using the Japanese Medical Data Vision claims database, 50.0% of patients were aged 65 years and older.^13^ In contrast, the proportion of the patients increased gradually and the peak proportions of both male and female patients were 51–60‐year‐old group in a study from Brazil.^16^ There may be more elderly patients than young patients in Japan. Moreover, there were more elderly patients with pustular psoriasis than in the past JSPR surveys of psoriasis.^3^, ^4^, ^5^, ^6^

Regarding the age at onset, majority of patients were in the 40–49‐year‐old group (15.8%) and the 60–69‐year‐old group (15.1%), followed by the 20–29‐year‐old group (14.0%) (Figure 2). The most common age of onset of the male patients was 60–69 years (19.9%), followed by 40–49 years (18.4%), while that of the female patients was 20–29 years (19.0%). In younger generations between 10–29 years, female patients were more than twice as many as male patients. Taken together, the age of onset of female patients was lower than that of male patients. This may be partly because pregnancy is associated with the onset of pustular psoriasis.

With respect to severity, in 57.0% of the patients, >20% of the BSA was affected (males, 60.1%; females, 54.2%), and many severe patients were registered in the present survey. The most common type was von Zumbusch type (59.8%; males, 61.6%; females, 58.2%).

A family history of psoriasis is often observed, and past JSPR surveys revealed that approximately 4.4%–6.4% of the patients with psoriasis had a family history.^3^, ^4^, ^5^, ^6^ In GPP, it is well known that interleukin‐36 receptor antagonist (IL36RN) and caspase recruitment domain family member 14 (CARD14) mutations are associated with the development of the disease.^17^ Although the presence or absence of these mutations was not analyzed in the present survey, a family history of psoriasis and PPP was observed in 4.1% and 1.7% of the patients, respectively. In a Japanese multicenter observational study, a family history of psoriasis was observed in 7.8% of the patients,^9^ which was 14.5% in a retrospective study in China^14^ and 31.2% in a retrospective study in Malaysia.^15^ Compared with these studies, the percentage was very low in the present survey.

Psoriasis is strongly associated with metabolic syndrome and cardiovascular diseases.^18^ In a past survey of patients with psoriasis, past history and comorbidities included hypertension (42.0%), dyslipidemia (30.0%), diabetes mellitus (23.7%), hyperuricemia (15.1%), cardiovascular disease (6.0%), and cerebrovascular disease (6.0%).^6^ In a Japanese multicenter observational study, the patients' comorbidities included hypertension (31.4%), dyslipidemia (8.8%), diabetes mellitus (14.7%), hyperuricemia (3.9%), cardiovascular disease (2.9%), and cerebrovascular disease (3.9%) in patients with pustular psoriasis.^9^ In the present survey, 67.4% of the patients had past history and comorbidities and there were more male patients than female patients. The patients' medical history and comorbidities included hypertension (39.8%), dyslipidemia (24.0%), diabetes mellitus (23.0%), hyperuricemia (6.6%), cardiovascular disease (7.1%), and cerebrovascular disease (7.7%). Hyperuricemia was not observed in the female patients. The percentages were higher in the present study, possibly due to the differences in patient characteristics and severity.

Regarding exacerbating factors, 27.5% of the patients had some type of exacerbating factor. Infection was the most common cause (26.3%), followed by stress (25.0%), certain drugs (22.5%), and pregnancy (15.0%). Among the female patients, pregnancy was the most common (27.3%). In the past JSPR surveys of patients with psoriasis, stress was the most common, followed by certain seasons and infection.^3^, ^4^, ^5^, ^6^ The exacerbating factors differ between the subtypes of psoriasis. Pustular psoriasis often occurs in patients administered corticosteroids. However, exacerbation caused by oral prednisolone was observed in only four patients.

In the present survey, 11.7% of the patients had some kind of focal infection. Odontogenic infection was the most common (52.9%), followed by tonsillitis (20.6%) and sinusitis (11.8%). Although the association between PPP and odontogenic infection is well established, odontogenic infection was frequently observed in patients with pustular psoriasis in the present survey.

In the present study, the most common region affected was the lower extremities (83.8%), followed by the abdomen (78.4%), upper extremities (76.3%), back (74.6%), and chest (72.2%) (Figure 3). There were no notable differences between male and female patients. In patients with psoriasis, the most common regions are the lower extremities, upper extremities, back, and scalp.^3^, ^4^, ^5^, ^6^ In contrast, the most common regions seem to be trunk and extremities in patients with pustular psoriasis. In the present survey, 2.1% of the patients had tongue lesions, which are characteristic of pustular psoriasis, but not PsV or PsA.

In the treatment group, 77.3% of the patients received topical therapy that included corticosteroids (67.1%), vitamin D3 (21.8%), and combinations of corticosteroid/vitamin D3 (43.6%). Topical corticosteroids were the most common. In contrast, 28 patients (9.6%) received phototherapy, of which 21 received NB‐UVB. Phototherapy is not commonly used in the treatment of pustular psoriasis. In terms of systemic therapy, 58.4% of the patients were treated with oral medications and 44.0% were treated with biologics. The most common oral medication was etretinate (52.4%), followed by corticosteroids (24.7%) and cyclosporin (22.9%). The most common biologic agent was ixekizumab (28.1%), followed by secukinumab (22.7%), infliximab (15.6%), guselkumab (14.8%), and risankizumab (10.2%). The top two were IL‐17A inhibitors. Bioswitch was observed in 22.7% of patients, which was common among infliximab, adalimumab, secukinumab, and ixekizumab. In contrast, a total of 27 patients (9.3%) received GMA, and GMA was more common during the observation period in pustular psoriasis than in PsA. Although the most common therapy varied among the studies,^9^, ^11^ the treatment trend remained unchanged during 2017–2020, in the present survey.

In conclusion, the present study evaluated data from annual epidemiological surveys of patients with pustular psoriasis from 2017 to 2020. This retrospective study did not include all Japanese patients with pustular psoriasis. However, the results will provide new and significant information regarding the recent perspective and trends of pustular psoriasis in the Japanese society.

## CONFLICT OF INTEREST

M. O. has received a grant for research and/or honoraria for lectures and/or advisory membership participation from Abbvie, Celgene, Eisai, Eli Lilly, Janssen, LEO Pharma, Maruho, Mitsubishi Tanabe Pharma, Novartis, Taiho Pharmaceutical, and Torii Pharmaceutical.

## References

[1] Griffiths CEM , Armstrong AW , Gudjonsson JE , Barker JNWN . Psoriasis. Lancet. 2021;397:1301–15. 10.1016/S0140-6736(20)32549-6 33812489

[2] Kamiya K , Kishimoto M , Sugai J , Komine M , Ohtsuki M . Risk factors for the development of psoriasis. Int J Mol Sci. 2019;20:4347. 10.3390/ijms20184347 31491865 PMC6769762

[3] Kawada A , Tezuka T , Nakamizo Y , Kimura H , Nakagawa H , Ohkido M , et al. A survey of psoriasis patients in Japan from 1982 to 2001. J Dermatol Sci. 2003;31:59–64. 10.1016/s0923-1811(02)00142-1 12615365

[4] Takahashi H , Nakamura K , Kaneko F , Nakagawa H , Iizuka H . Analysis of psoriasis patients registered with the Japanese Society for Psoriasis Research from 2002‐2008. J Dermatol. 2011;38:1125–9. 10.1111/j.1346-8138.2010.01145.x 21951304

[5] Ito T , Takahashi H , Kawada A , Iizuka H , Nakagawa H . Epidemiological survey from 2009 to 2012 of psoriatic patients in Japanese Society for Psoriasis Research. J Dermatol. 2018;45:293–301. 10.1111/1346-8138.14105 29115687

[6] Kamiya K , Oiso N , Kawada A , Ohtsuki M . Epidemiological survey of the psoriasis patients in the Japanese Society for Psoriasis Research from 2013 to 2018. J Dermatol. 2021;48:864–75. 10.1111/1346-8138.15803 33580908 PMC8247979

[7] Gooderham MJ , Van Voorhees AS , Lebwohl MG . An update on generalized pustular psoriasis. Expert Rev Clin Immunol. 2019;15:907–19. 10.1080/1744666X.2019.1648209 31486687

[8] Ohkawara A , Yasuda H , Kobayashi H , Inaba Y , Ogawa H , Hashimoto I , et al. Generalized pustular psoriasis in Japan: two distinct groups formed by differences in symptoms and genetic background. Acta Derm Venereol. 1996;76:68–71. 10.2340/00015555766871 8721499

[9] Ohata C , Tsuruta N , Yonekura K , Higashi Y , Saito K , Katayama E , et al. Clinical characteristics of Japanese pustular psoriasis: a multicenter observational study. J Dermatol. 2022;49:142–50. 10.1111/1346-8138.16217 34723399

[10] Ozawa A , Ohkido M , Haruki Y , Kobayashi H , Ohkawara A , Ohno Y , et al. Treatments of generalized pustular psoriasis: a multicenter study in Japan. J Dermatol. 1999;26:141–9. 10.1111/j.1346-8138.1999.tb03444.x 10209919

[11] Miyachi H , Konishi T , Kumazawa R , Matsui H , Shimizu S , Fushimi K , et al. Treatments and outcomes of generalized pustular psoriasis: a cohort of 1516 patients in a nationwide inpatient database in Japan. J Am Acad Dermatol. 2022;86:1266–74. 10.1016/j.jaad.2021.06.008 34116101

[12] Fujita H , Terui T , Hayama K , Akiyama M , Ikeda S , Mabuchi T , et al. Japanese guidelines for the management and treatment of generalized pustular psoriasis: the new pathogenesis and treatment of GPP. J Dermatol. 2018;45:1235–70. 10.1111/1346-8138.14523 30230572

[13] Morita A , Kotowsky N , Gao R , Shimizu R , Okubo Y . Patient characteristics and burden of disease in Japanese patients with generalized pustular psoriasis: results from the medical data vision claims database. J Dermatol. 2021;48:1463–73. 10.1111/1346-8138.16022 34212422 PMC9291902

[14] Zheng J , Chen W , Gao Y , Chen F , Yu N , Ding Y , et al. Clinical analysis of generalized pustular psoriasis in Chinese patients: a retrospective study of 110 patients. J Dermatol. 2021;48:1336–42. 10.1111/1346-8138.15958 34018629 PMC8453703

[15] Choon SE , Lai NM , Mohammad NA , Nanu NM , Tey KE , Chew SF , et al. Clinical profile, morbidity, and outcome of adult‐onset generalized pustular psoriasis: analysis of 102 cases seen in a tertiary hospital in Johor. Malaysia Int J Dermatol. 2014;53:676–84. 10.1111/ijd.12070 23967807

[16] Duarte GV , Esteves de Carvalho AV , Romiti R , Gaspar A , Gomes de Melo T , Soares CP , et al. Generalized pustular psoriasis in Brazil: a public claims database study. JAAD Int. 2022;6:61–7. 10.1016/j.jdin.2021.12.001 35059660 PMC8760386

[17] Sugiura K . The genetic background of generalized pustular psoriasis: IL36RN mutations and CARD14 gain‐of‐function variants. J Dermatol Sci. 2014;74:187–92. 10.1016/j.jdermsci.2014.02.006 24656634

[18] Arida A , Protogerou AD , Kitas GD , Sfikakis PP . Systemic inflammatory response and atherosclerosis: the paradigm of chronic inflammatory rheumatic diseases. Int J Mol Sci. 2018;19:1890. 10.3390/ijms19071890 29954107 PMC6073407

